# Immunization with different recombinant West Nile virus envelope proteins induces varying levels of serological cross-reactivity and protection from infection

**DOI:** 10.3389/fcimb.2023.1279147

**Published:** 2023-11-15

**Authors:** Rebecca Weiß, Leila Issmail, Alexandra Rockstroh, Thomas Grunwald, Jasmin Fertey, Sebastian Ulbert

**Affiliations:** Fraunhofer Institute for Cell Therapy and Immunology, Department of Vaccines and Infection Models, Leipzig, Germany

**Keywords:** West Nile virus, vaccine, recombinant proteins, fusion loop, cross-reactivity, flavivirus

## Abstract

**Introduction:**

West Nile Virus (WNV) is a zoonotic flavivirus transmitted by mosquitoes. Especially in the elderly or in immunocompromised individuals an infection with WNV can lead to severe neurological symptoms. To date, no human vaccine against WNV is available. The Envelope (E) protein, located at the surface of flaviviruses, is involved in the invasion into host cells and is the major target for neutralizing antibodies and therefore central to vaccine development. Due to their close genetic and structural relationship, flaviviruses share highly conserved epitopes, such as the fusion loop domain (FL) in the E protein, that are recognized by cross-reactive antibodies. These antibodies can lead to enhancement of infection with heterologous flaviviruses, which is a major concern for potential vaccines in areas with co-circulation of different flaviviruses, e.g. Dengue or Zika viruses.

**Material:**

To reduce the potential of inducing cross-reactive antibodies, we performed an immunization study in mice using WNV E proteins with either wild type sequence or a mutated FL, and WNV E domain III which does not contain the FL at all.

**Results and discussion:**

Our data show that all antigens induce high levels of WNV-binding antibodies. However, the level of protection against WNV varied, with the wildtype E protein inducing full, the other antigens only partial protection. On the other hand, serological cross-reactivity to heterologous flaviviruses was significantly reduced after immunization with the mutated E protein or domain III as compared to the wild type version. These results have indications for choosing antigens with the optimal specificity and efficacy in WNV vaccine development.

## 1 Introduction

The human pathogenic and mosquito-transmitted West-Nile Virus (WNV), belongs to the family *Flaviviridae*, which are enveloped viruses that contain a single stranded, positive sense RNA genome with a length of 11kb. It encodes three structural proteins (Capsid protein, Envelope protein, Premembrane protein) which form the virion, and seven nonstructural proteins, which are translated as a single polyprotein that is co- and post-translationally cleaved by viral and host proteases. (Hayes et al., 2005; Welsch et al., 2009). Flaviviridae also include a number of other human pathogens, such as Dengue (DENV), Japanese encephalitis virus (JEV), Zika (ZIKV), Tick-borne encephalitis (TBEV) or Yellow fever (YFV) viruses. WNV is currently the most widely spread vector-borne flavivirus (Chancey et al., 2015) and is endemic in regions of the Americas (Hadfield et al., 2019), Europe (Giesen et al., 2023), Africa (García-Carrasco et al., 2023), Australia (Prow et al., 2016) and Asia (Bassal et al., 2017). WNV primarily circulates between birds, the natural reservoir, and mosquitoes (Kilpatrick et al., 2007). The vectors can transmit WNV also to mammals including horses and humans, however, these represent dead-end hosts and do not re-infect mosquitoes (Sewgobind et al., 2023). Nevertheless, transmission between humans may occur via organ or blood transplantation (Centers for Disease Control and Prevention (CDC), 2009; Byas and Ebel, 2020).

Infections in humans mainly lead to mild, flu-like symptoms or remain asymptomatic (Hayes et al., 2005). However, approx. 1% of infections are affecting the central nervous system (CNS) causing encephalitis, meningitis and muscle paralysis with a possible fatal outcome (Bai et al., 2019). The risk for severe, neuroinvasive WNV infections increases with age (Montgomery, 2016) or is correlated to an immunocompromised immune system (Sejvar, 2016). To date, there is no specific treatment for WNV disease, hence clinical management is purely supportive. An infection with WNV leads to a long-lasting immunity to the virus. Several vaccine approaches have been developed for the prevention of WNV induced disease in humans, although until now none has progressed beyond phase 2 clinical studies (Gould et al., 2023).

The key antigen in almost all WNV vaccine candidates is the envelope (E) protein, which is targeted by a variety of virus neutralizing antibodies. These antibodies are critical for the induction of a protective immunity (Pierson et al., 2008). The E protein consists of an ectodomain that is anchored in the viral envelope and is divided into the major domains I, II, and III. Domain II (EDII) contains the fusion loop (FL), and domain III (EDIII) binds to the host cell receptor(s) (Zhang et al., 2017). For immunization studies, the E protein has been administered in different forms: as recombinant protein, as part of virus-like particles (VLP) or via inactivated viruses and via different viral vectors, and in general, protective immune responses were induced (Davis et al., 2001; Bonafé et al., 2009; Lieberman et al., 2009; Brandler et al., 2012; Dayan et al., 2013; Pinto et al., 2013; Magnusson et al., 2014). However, the close genetic and structural relationship of flaviviruses leads to highly conserved epitopes within the E protein, and as a consequence a major problem in flavivirus immunology remains the cross-reactivity of immune responses, especially the induction of cross-reacting antibodies (Rey et al., 2018). Such cross-reacting antibodies have been linked to the phenomenon of antibody dependent enhancement of infection (ADE). Although not yet understood in detail, ADE was associated to non- or sub-neutralizing antibodies that bind the virus and can lead to increased viral entry into host cells due to Fc-receptor mediated endocytosis (Sarker et al., 2023). ADE is most problematic in areas where closely related flaviviruses co-circulate and has been documented clinically between infections of the different DENV serotypes, but also in subsequent infections of ZIKV and DENV (Rothman, 2011; Katzelnick et al., 2017; Katzelnick et al., 2020). In addition, ADE between different flaviviruses has been demonstrated in preclinical models, e.g. WNV antibodies enhanced the infection with ZIKV (Bardina et al., 2017). Importantly, ADE is of concern for the development of flavivirus vaccines, as vaccine-induced antibodies might constitute a risk for enhanced disease upon infection with another flavivirus or between different serotypes within one flavivirus (Halstead, 2018; Ulbert, 2019).

A large proportion of cross-reactive flavivirus antibodies target the highly conserved fusion loop (FL) of the E protein, which contains stretches of amino acids that are almost identical in many human pathogenic flaviviruses (Crill and Chang, 2004; Rey et al., 2018). Modifying the FL by mutations or eliminating the FL by using only EDIII leads to greatly reduced binding of cross-reactive antibodies from human infections (Roberson et al., 2007; Rockstroh et al., 2019). Using FL-deleted or FL-mutated VLPs of ZIKV, the induction of ADE was decreased in animal models, but a decrease in efficacy was also reported (Richner et al., 2017; Yang et al., 2017).

The recombinant WNV E protein is one of the few vaccine candidates that have entered early phase clinical testing (Kaaijk and Luytjes, 2018). We therefore tested a recombinant E protein with four mutations in and near the FL (termed Equad protein) as a vaccine antigen to prevent WNV induced disease in a mouse model. The induction of cross-reactive flavivirus antibodies and the protective efficacy were compared to the wildtype version of E (Ewt) and to recombinant EDIII.

## 2 Results

### 2.1 Analysis of recombinant vaccine antigens

**Figure 1:**
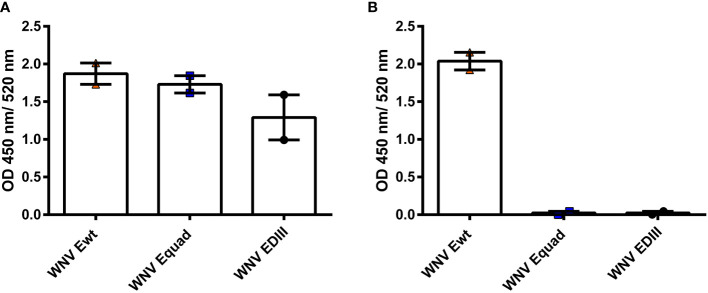
Structural assessment of recombinant WNV proteins with monoclonal antibodies. ELISAs with anti-WNV antibody E16, recognizing an epitope within EDIII **(A)** and anti-flavivirus antibody 4G2, recognizing the FL **(B)** to recombinant WNV E proteins (200 ng protein coating per microtiter well). The Data derive from two independent measurements with each sample measured in duplicates. Indicated are mean values ± standard error (SEM) for each group.

We performed an immunization study in mice using recombinant WNV ectodomains of E proteins having either the wildtype sequence (Ewt) or four mutations in or near the FL (Equad). Additionally, the domain III (EDIII) was included. Ewt and Equad were expressed in *Drosophila* S2 cells and EDIII in *E. coli.* To analyze the structural conformation of the proteins before immunization, serological analysis was carried out by ELISA. A monoclonal antibody (mAb) binding to an epitope at the lateral ridge of domain III with virus neutralizing activity (Oliphant et al., 2006) comparably recognized Ewt, Equad and to a slightly lesser extend EDIII (**Figure 1A**). The proportion of protein recognized by the mAb E16 was lower on EDIII, considering that equal amounts of protein were coated and EDIII has a smaller molecular weight than Ewt and Equad (indicating a potential loss of correctly folded EDIII in the bacterial expression system). Analysis using the FL-specific monoclonal antibody 4G2 (Nawa et al., 2001; Crill and Chang, 2004) confirmed that the FL is mutated or absent in Equad and EDIII, respectively (**Figure 1B**).

### 2.2 Humoral immune response

**Figure 2:**
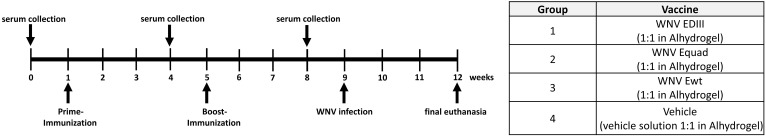
Immunization experiment and serum collection. Female BALB/c mice (n = 6 per group) were immunized two times with variants of the recombinant West Nile virus envelope Protein (WNV E) or with vehicle solution with adjuvant (Vehicle) as control.

**Figure 3:**
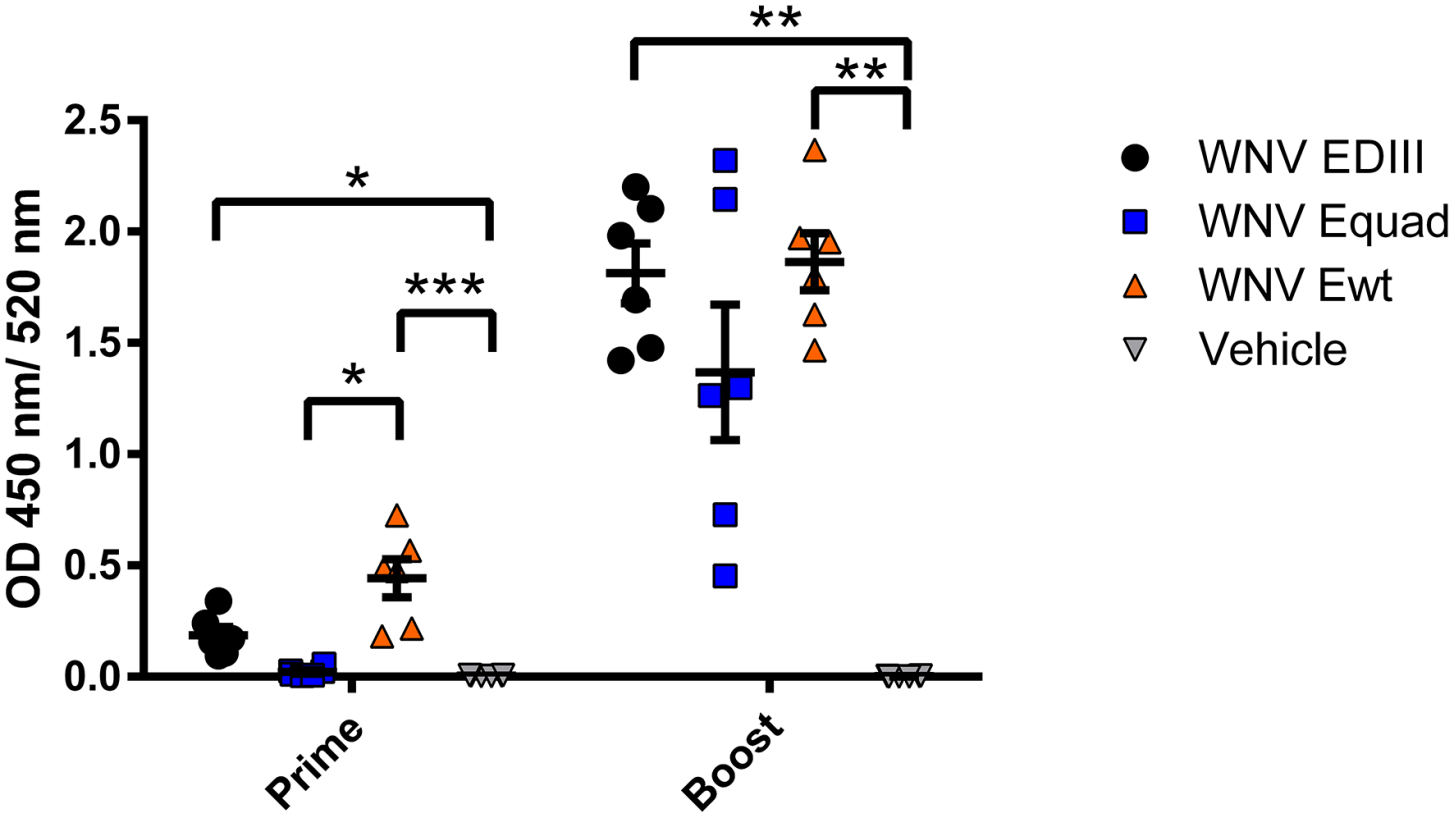
Humoral immune responses after vaccination with variants of the recombinant WNV envelope protein and vehicle immunization. Binding of serum antibodies three weeks after prime and boost immunization was measured in an IgG-ELISA using homologous recombinant proteins. Each data point represents the mean values of one individual mouse from two independent measurements with each sample measured in duplicates. Indicated are mean values ± standard error (SEM) for each group. The data were analyzed with Kruskal-Wallis Test followed by Dunn’s multiple comparisons test (*p<0.05; **p<0.01; ***p<0.001).

Mice were immunized two times with a four weeks interval with 20 µg of either Ewt, Equad, EDIII or buffer (vehicle control), all with Alhydrogel as adjuvant (**Figure 2**). Using ELISAs with the respective antigen, animals were assessed for the induction of a specific IgG response post vaccination (**Figure 3**). After the prime immunization mice vaccinated with WNV Ewt displayed the highest signals. After boost immunization, all three groups showed high titers of binding antibodies, and the titers induced by Equad were more heterogenous than in the other groups. In contrast, no antibodies were detectable in the control group measured on Ewt protein (which includes the protein sequence of EDIII) as coating antigen.

**Figure 4:**
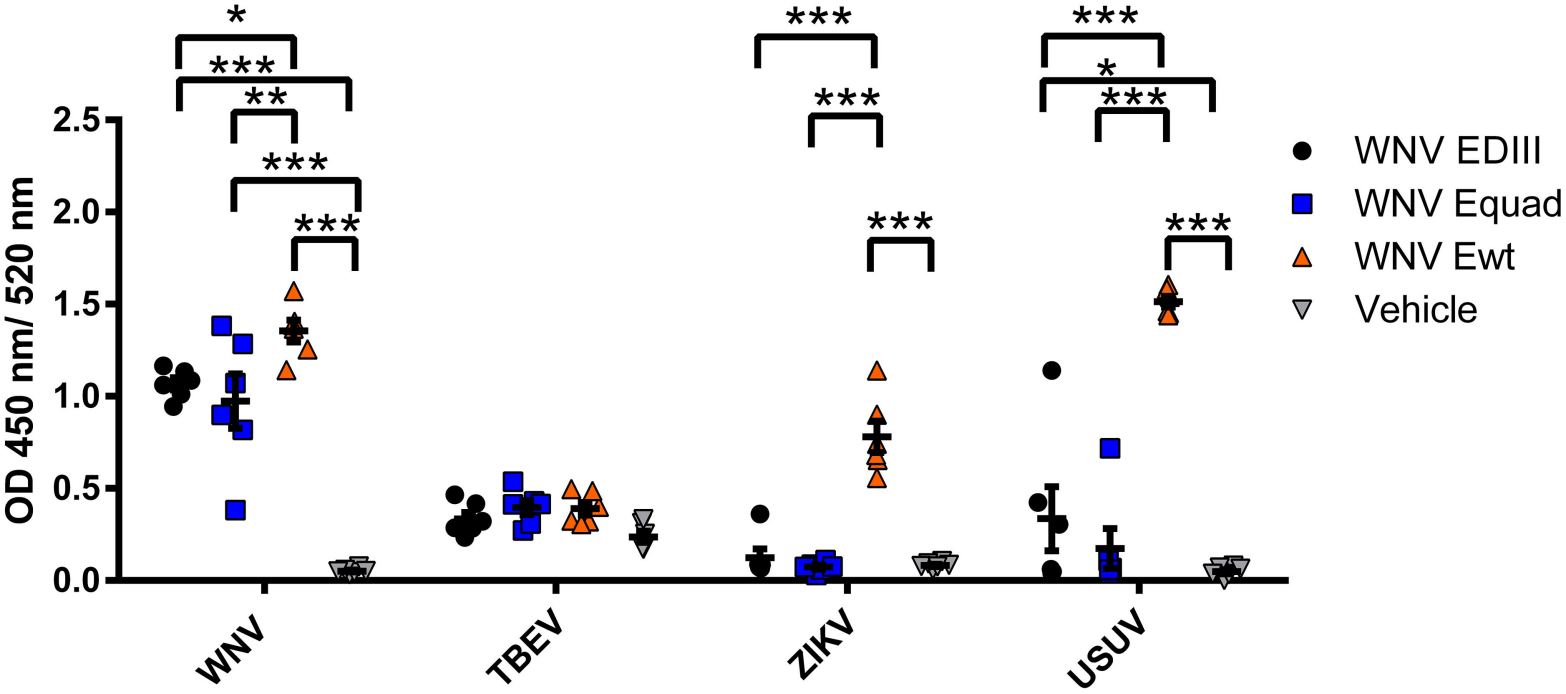
Cross-reactivity of mouse sera after vaccination with variants of the recombinant WNV envelope protein and vehicle immunization. Binding of serum antibodies (three weeks after boost vaccination) to full virions of flaviviruses was analyzed in an IgG-ELISA using purified TBEV, ZIKV, USUV and WNV as coating antigens. Each data point represents the mean values of one individual mouse from two independent measurements with each sample measured in duplicates. Indicated are mean values ± standard error (SEM) for each group. The data were analyzed with RM two-way ANOVA followed by Dunn’s multiple comparisons test (*p<0.05; **p<0.01; ***p<0.001).

To determine the cross-reactivity of serum antibodies after boost immunization, sera were analyzed using whole virions of WNV or the related flaviviruses TBEV, ZIKV and Usutu virus (USUV) (**Figure 4**). Antibodies from all three immunized groups bound to WNV. For TBEV, only minimal antibody binding was detected, with no statistically significant differences between the protein immunized groups and the control group. When tested on ZIKV the signals of EDIII and Equad immunized mice were in the range of the control group. In contrast, sera from the WNV Ewt group showed a high signal on ZIKV, significantly higher than the Equad or EDIII groups. A similar pattern was observed for binding to USUV. This suggests that immunization with Ewt induces a higher amount of cross-reacting flavivirus antibodies compared to Equad or EDIII.

### 2.3 *In vivo* protection assay

**Figure 5:**
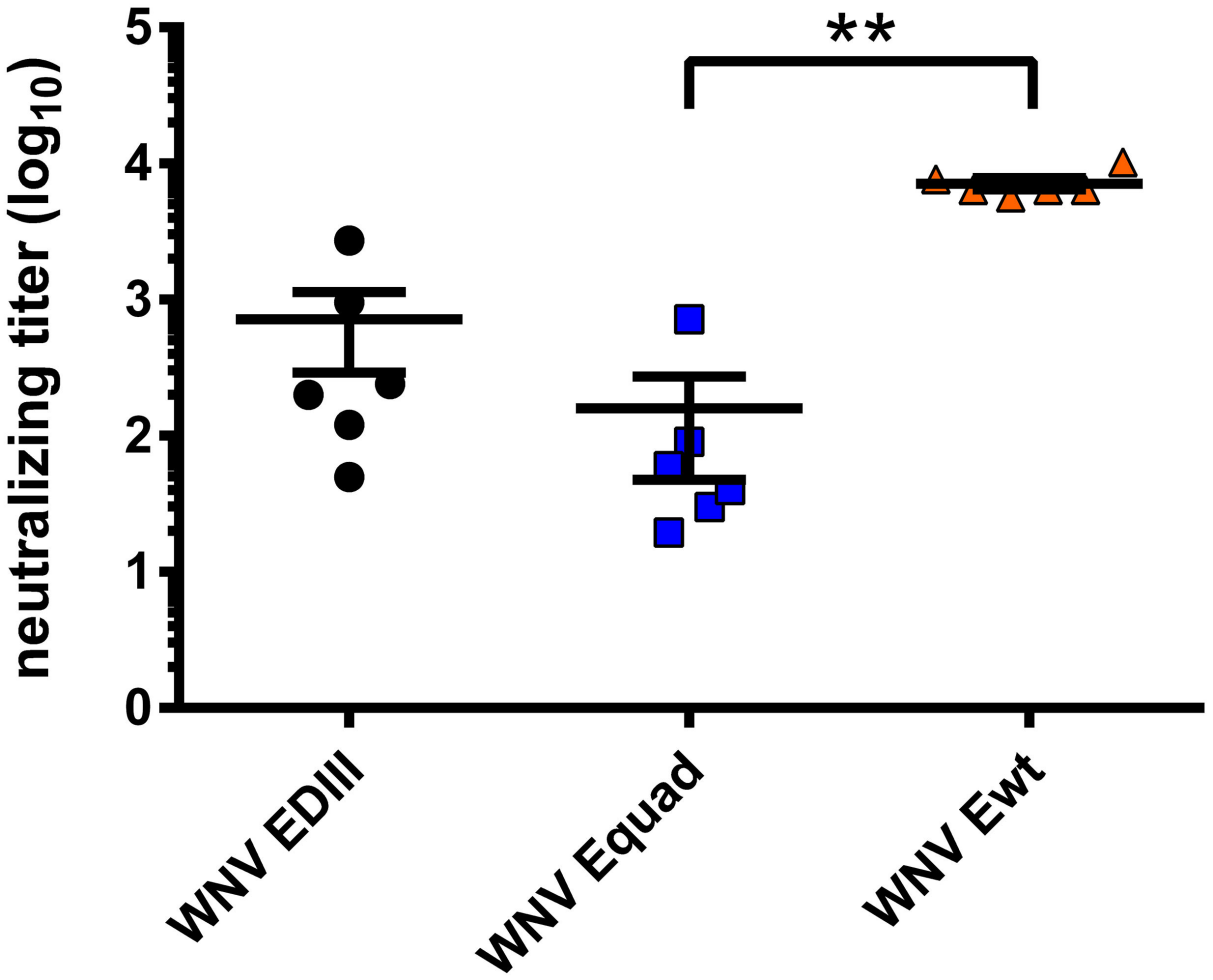
WNV-neutralizing capacity of mouse sera *in vitro*. Neutralizing antibody titers of sera three weeks after boost vaccination were measured for each mouse through focus reduction neutralization tests (FRNT_50_). Each data point equals the mean value for every individual mouse serum from two independent measurements. Shown are mean ± standard error (SEM) for each group. The data were analyzed with Kruskal-Wallis Test followed by Dunn’s multiple comparisons test (**p<0.01).

Next, the protective efficacy of the induced antibodies was analyzed by cellular virus neutralization tests (**Figure 5**). Vaccination with EDIII and Equad elicited neutralizing titers at a mean of 715 and 160, respectively. However, immunization with Ewt induced a mean neutralizing titer of 7,131, which was significantly and more than 10-fold higher than those obtained with Equad. No neutralizing antibodies were detectable in the vehicle control group.

**Figure 6:**
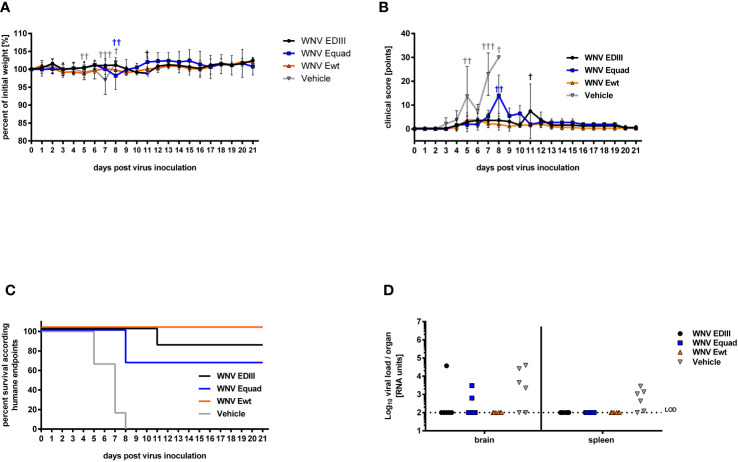
Protective efficacy of protein-based vaccine in mice against lethal WNV infection. Four weeks after boost immunization, female BALB/c mice (n = 6) were infected with a lethal dose of West Nile virus via intraperitoneal injection. All animals were monitored daily for body weight **(A)** and clinical score **(B)**. Animals reaching humane endpoints were euthanized and are marked by a cross (†). Data are presented as means ± standard errors. The percentages of surviving animals according to humane endpoints are shown **(C)**. Overlapping lines are partially offset for better readability. After euthanasia, brain and spleen tissues were homogenized and their viral load was quantified by RT-qPCR. Shown are viral RNA units of individual animals **(D)**. Limit of detection (LOD) is indicated by a dotted line.

To assess protection from lethal infection, the weight (**Figure 6A**), clinical score (**Figure 6B**) and survival (**Figure 6C**), of immunized mice were monitored for three weeks after challenge with WNV strain Ita09 (10^4^ FFU/mouse) and viral load in brain and spleen was assessed by RT-qPCR (**Figure 6D**). All animals in the vehicle control group had to be euthanized according to humane endpoints between day 5 and day 8 post infection. Four animals in the control group had detectable viral RNA in the brain and five in the spleen. While all animals that were immunized with Ewt survived, two animals in the Equad group and one animal in the EDIII group succumbed to the infection. In those animals, viral RNA was detected in the brain, but not in the spleen. None of the surviving mice had detectable WNV RNA in the organs investigated. The differences in survival were statistically significant between the vehicle control group and the groups immunized with Ewt (p<0.001), EDIII (p<0.001) and Equad (p<0.01).

## 3 Discussion

Vaccine development against WNV focuses on the E protein, which plays multiple critical roles in the viral life cycle and is therefore considered the primary target for neutralizing antibodies. We conducted an immunization study using different recombinantly expressed versions of the WNV E protein to investigate the role of the FL domain in cross-reactivity and efficacy. Mice were vaccinated in a homologous prime-boost regime with the full E ectodomain either in its wildtype form or with a mutated FL, or with EDIII, which completely lacks the FL. IgG antibody titers to the homologous protein antigens remained low after prime, except for Ewt, but strongly increased after the boost immunization. These antibodies also recognized WNV virions.

When analyzing the cross-reactivity of the antibodies to other flaviviruses, the differences between the groups were significant. Mice immunized with Ewt displayed high antibody titers against ZIKV and USUV, whereas the Equad or EDIII immunized groups showed no or only minimal cross-reactive signals. This antibody cross-reactivity induced by Ewt was in accordance with the phylogenetic relationship of the analyzed flaviviruses: it was most prominent with USUV, the flavivirus most closely related to WNV and part of the same JEV serocomplex, followed by ZIKV (Dutta and Langenburg, 2023). No cross-reactivity was observed with TBEV, which is most distantly related to WNV. These results confirm the critical role of the FL in the induction of cross-reactive immune responses (Rey et al., 2018). In WNV serology, a modified FL has been demonstrated to abolish the binding of antibodies induced by heterologous flavivirus infections (Chabierski et al., 2014). In addition, FL-mutated E proteins have been used as parts of virus-like particles (VLPs) in vaccine development against ZIKV, DENV and JEV, resulting in a strong reduction of cross-reactive antibodies (Crill et al., 2012; Richner et al., 2017; Kotaki et al., 2022). In contrast to these previous vaccine studies, we have used recombinant E ectodomains rather than VLPs, and demonstrate that the WNV E protein with mutated FL significantly reduces the induction of antibodies to heterologous flaviviruses.

The phenomenon of ADE due to cross-reactivity of flavivirus-induced immune responses is best known from infections with different DENV serotypes, although the exact molecular mechanism is still not fully understood (Rothman, 2011). Recently, evidence of ADE was also reported from infections with DENV and ZIKV (Katzelnick et al., 2020). It remains uncertain to what extent cross-reactive antibodies induced by WNV infection or vaccination could lead to similar problems. While there is reported evidence that sera from WNV infected individuals can enhance ZIKV infections *in vitro* and *in vivo* (Bardina et al., 2017), this question has not yet been addressed in clinical studies. The continuous spread of WNV into areas where USUV is circulating, e.g. in Italy, increases the likelihood of subsequent infections, and to date neither cross-protection nor enhancement of infections can be excluded (Sinigaglia et al., 2019). In addition, the (re-) emergence of yet unknown or so far neglected flaviviruses should be considered when assessing the safety of flavivirus vaccines. As a prominent example, ZIKV had not been considered a major problem until its emergence in Micronesia (Duffy et al., 2009). To date, the virus is globally spread, and coinfections with DENV are commonly observed (Rodriguez-Barraquer et al., 2019). The development of WNV vaccines should therefore address the phenomenon of antibody cross-reactivity, even though there is currently no clinical evidence supporting ADE due to WNV infections.

When the immunized animals were analyzed for the induction of protective immune responses, clear differences were observed between Ewt on one side, and Equad and EDIII on the other. Ewt resulted in high titers of virus neutralizing antibodies, whereas lower titers were measured in the Equad and EDIII groups. These lower titers of neutralizing antibodies do only poorly correspond to the higher levels of binding antibodies induced by the immunizations. This reflects the finding that neutralizing antibodies only constitute a minor fraction of the humoral immune response to WNV antigens (Throsby et al., 2006). Likewise, all animals immunized with Ewt survived a lethal WNV infection, whereas two and one animals immunized with Equad or EDIII, respectively, did not. Neutralizing antibodies were induced in all immunized animals, but animals succumbing to the infection did not display the lowest titers in the individual groups. This indicates that neutralizing antibodies alone were poorly predictable for protection in the chosen immunization setup. The control animals had WNV RNA detectable in brain and spleen. In contrast, the immunized mice that did not survive the challenge had WNV RNA detectable only in the brain, suggesting a reduction of viral spreading due to the vaccines.

This study represents the first investigation of a recombinant WNV E protein with mutated FL as a vaccine. Nevertheless, vaccination studies using ZIKV VLPs with E proteins containing a mutant FL also reported a decrease in protective capacity when compared to the wildtype version (Richner et al., 2017; Thompson et al., 2022). These studies suggested impaired structures of the VLP due to the FL mutations, leading to altered quaternary epitopes. Unlike these studies we used single E ectodomains rather than VLPs. Hence, alteration of critical epitopes due to FL mutations can only affect the E protein monomer. Interestingly, the binding of the neutralizing antibody E16, which recognizes an epitope at the lateral ridge of EDIII (Oliphant et al., 2007), remained unaffected by the FL mutations (**Figure 1**). Therefore, impairment or loss of neutralizing epitopes apparently affects other regions of the protein. This question could be addressed by a detailed scan of the Equad protein with monoclonal antibodies against structural epitopes. In addition, it cannot be excluded that the absence or mutation of the FL itself leads to a decrease in the overall protection from WNV, as neutralizing capacity of some FL antibodies has been described for flaviviruses, although with varying efficacy (Vogt et al., 2011; Dai et al., 2016).

A potential approach to increase the protective efficacy of FL-mutant E proteins might involve additional boost immunizations, as repeated boosting is well established to enhance and broaden the neutralizing antibody repertoire and protection (Burckhardt et al., 2022). In this study only one boost was administered, similar to the previous studies reporting a lower protective response with FL mutations (Richner et al., 2017; Thompson et al., 2022). However, we have previously demonstrated for an Equad protein of ZIKV that after three immunizations no differences were observed compared to the wildtype version in the induction of neutralizing antibodies (Berneck et al., 2020). Likewise, investigations using three doses of DNA vaccines coding for VLPs with a single point mutation in the FL reported no decrease in neutralizing antibodies for WNV or DENV when compared to the wildtype VLPs (Crill et al., 2012; Yamanaka et al., 2022).

Unlike the Equad protein, recombinant EDIII from different protein expression systems has been used in previous WNV vaccination studies. Depending on the exact study design, these investigations reported the induction of highly protective or only partially protective immune responses (Chu et al., 2007; Martina et al., 2008; Zlatkovic et al., 2011; Friedrich et al., 2016; Lai et al., 2018). Similar to the antigens with a mutant FL, immunization schedules including at least two boosters generally resulted in higher efficacy. Therefore, increasing the number of booster immunizations with Equad or EDIII might be a way to increase vaccine efficacy and at the same time minimize the induction of flavivirus cross-reactive immune responses.

In summary, our results confirm that the ectodomain of the WNV E protein is a potent vaccine antigen. However, its original sequence induces cross-reacting antibodies to related flaviviruses. Although there is no clinical evidence yet that pre-existing WNV immunity could lead to disease enhancement upon infection with known heterologous flaviviruses, this concern should be taken in account in WNV vaccine development. Our findings demonstrate that the Equad or the EDIII antigens induce significantly less cross-reactive antibodies than the wildtype E ectodomain. However, the efficacy of both antigens needs to be improved through optimization of immunization schedules before WNV vaccines based on these proteins can progress to clinical development.

## 4 Materials and methods

### 4.1 Cells and viruses

Vero E6 cells (DSMZ, Braunschweig, Germany) were propagated in Dulbecco’s Modified Eagle Medium (DMEM, Gibco, Carlsbad, USA) supplemented with 10% heat inactivated fetal calf serum (FCS, Gibco) and 1% penicillin/streptomycin (Gibco) at 37°C and 5% CO_2_.

Viruses used in this study were West Nile Virus (genetic lineage 1 strain WNV-Ita09, kindly provided by Luisa Barzon, Padova University), Zika Virus (Dominican Republic/2016/PD1, kindly provided by Luisa Barzon), Usutu Virus (strain 3345, isolate Arb276, provided by the European Viral Archive Global EVAg), Tick-borne encephalitis Virus (strain Hypr 9BMP U39292.1, kindly provided by Uwe Liebert, Leipzig University). Viruses were propagated in Vero E6 cells and purified from culture supernatant by ultracentrifugation. Virus titration in focus forming units (FFU) was performed as previously described (Berneck et al., 2020). In short, serial dilutions of virus were incubated on Vero E6 monolayers for 1 h at 37°C. After removal of the supernatant, cells were overlaid with 1% methylcellulose in DMEM supplemented with 2% FCS and 1% penicillin/streptomycin. Cells were fixed after 16 – 22 h with 4% formaldehyde (Roth, Karlsruhe, Germany) in phosphate buffered saline (PBS). Perm Wash buffer (0.1% BSA (Roth) and 0.1% Saponin (Roth) in PBS) was used for permeabilization, blockage and washing of cells. The primary anti-flavivirus antibody 4G2 (absolute antibody, Oxford, UK, 1:2,000), an anti-mouse IgG HRP-conjugated secondary antibody (Dako, Denmark, 1:1,500) and TrueBlue peroxidase substrate (SeraCare, Milford, USA) were used for immunostaining. Spots were counted automatically with an Immunospot Universal Analyzer (Cellular Technology Limit, Cleveland, USA).

For analyzing virus-binding antibodies, viruses were coated on ELISA plates (see below), except for WNV, which was inactivated before by overnight treatment with 0.3% H_2_O_2_ (Roth, Karlsruhe, Germany) in Sucrose-PBS at 37°C followed by dialysis against PBS.

### 4.2 Expression and purification of recombinant proteins

The wildtype and quadruple mutant E‐proteins (Equad) from WNV (isolate NY2000 ‐ crow3356, E‐protein amino acid residues 1‐400 bearing the mutations T76A, M77G, W101R, L107R) have been described previously (Rockstroh et al., 2019).

The expression vector encoding domain III of the WNV E-protein (isolate NY2000 ‐ crow3356 E‐protein amino acid residues 299-400) as a fusion protein with the maltose binding protein (MBP) was described previously (Schneeweiss et al., 2011). The fusion protein was expressed in *E. coli* strain Rosetta 2 DE3 (Merck, Darmstadt, Germany) by induction with 1 mM isopropyl-beta-D-thiogalactopyranoside (IPTG, Janssen Pharmaceuticals, Beerse, Belgium). Bacteria were harvested by centrifugation (4,500 x g, 15 min, 4°C) and lysed in buffer containing 20 mM Tris pH 7.4 (Roth), 200 mM NaCl (Roth), 1 mM EDTA, 10 mM Imidazol (Roth), 10% Glycerol (Roth), 1 mM DTT (PanReac Applichem ITW Reagents, Darmstadt, Germany) and protease-inhibitor (Sigma Aldrich, St. Louis, USA) using a high-pressure homogenizer (Constant Systems LTD, Daventry, UK). Insoluble cell debris was separated from the soluble fraction containing WNV EDIII by centrifugation (15,000 x g, 30 min, 4°C). The recombinant MBP-fusion protein was purified on Amylose resin columns (New England Biolabs, Frankfurt, Germany). Subsequently the MBP-tag was removed by Factor Xa Protease (New England Biolabs, Frankfurt, Germany) cleavage and the resulting WNV EDIII His-tagged protein was further purified on His60 Ni Superflow Resin columns (TaKaRa Bio, USA). The protein was then dialyzed against PBS, and aliquots were stored at - 80°C until use.

Bradford assay and SDS-gel electrophoresis were performed for measuring protein amount and assessment of protein purity, respectively (data not shown).

### 4.3 Mouse immunization and challenge experiment

The mouse experiment was carried out in accordance with the EU Directive 2010/63/EU for animal experiments and was approved by local authorities (Landesdirektion Sachsen). Female 8-week-old BALB/c mice were purchased from Charles River (Sulzfeld, Germany) and randomly assigned into groups of 6 mice. Mice were housed in a specific pathogen-free environment in individually ventilated cages, 12 h/12 h-light/dark cycle, and water and mouse chow were provided ad libitum.

For vaccine preparation, purified proteins (EDIII, Equad, and Ewt) were diluted in PBS before mixing gently with Alhydrogel (aluminum hydroxide gel adjuvant, 10 mg/mL aluminum, InvivoGen, Toulouse, France) at a 1:1 volume ratio to administer 20 µg protein in 100 µL per mouse. The adjuvant was chosen based on the wide experience available with aluminium hydroxide and recombinant protein immunizations (Zlatkovic et al., 2011). For the immunization, inhaled light isoflurane anesthesia was applied, and each vaccine was injected in the musculus gastrocnemius of each hind limb (50 µL each). Control mice were sham-immunized with vehicle solution (1:1 mixture of Alhydrogel and PBS). All mice were immunized twice at four-week interval.

Blood was sampled from the retro-bulbar venous sinus one week prior to the prime and boost immunization, as well as one week before the challenge. Collected blood samples were incubated at room temperature for 30 min and centrifuged at 8,000 x g for 10 min to obtain serum for antibody analysis. Four weeks after the boost immunization, mice were challenged by intraperitoneal (i.p.) injection of 10^4^ FFU of purified WNV-Ita09 in a total volume of 100 µL per mouse. The infection and all following work were carried out under biosafety level 3 (BSL3) conditions. Clinical development of disease was monitored daily for 21 days post-infection and score points were given according to the following criteria: body weight loss (0 points= no weight loss, 5 points= 8-10%, 10 points= 11-19%, 20 points= weight loss ≥20% of initial weight); fur condition (0 points = shiny and clean coat, 2 points = piloerection, 5 points = ruffled fur); eye appearance (0 points= open healthy eyes, 5 points= mildly inflamed, 10 points = highly inflamed and closed); gastrointestinal symptoms due to distention of the intestine (0 points= no symptoms, 5 points= mild, 10 points= moderate abdominal swelling); body posture (0 points= normal posture, 20 points= hunched body posture); activity level and motor function (5 points= slightly reduced activity and reaction, 10 points= coordination disorder and reduced activity, 20 points= apathy and morbidity). Score points of 0-9 were defined as mild, 10-19 as moderate, and ≥20 as severe. Humane endpoints requiring euthanasia were defined as reaching a cumulative score points of 20 for a period of 24 h. Animals acquiring cumulative score points greater than 20 were immediately euthanized. Surviving mice were euthanized 21 days after the challenge.

Spleens and brains were isolated and homogenized in gentleMACS™ M Tubes (Miltenyi Biotec, Bergisch Gladbach, Germany) containing 2 mL of ice-cold PBS using gentleMACS Dissociator (Miltenyi Biotec, Bergisch Gladbach, Germany). Homogenized tissues were cleared of cell debris by centrifugation for 5 min at 2,000 × g and 4°C. Homogenates were stored at - 80°C until viral isolation.

### 4.4 Viral RNA extraction and RT-qPCR

Viral RNA was isolated from 140 μL cell-free supernatant of organ homogenate using QIAamp-Viral-RNA-Mini Kit (Qiagen, Hilden, Germany) according to the manufacturer’s instructions. Briefly, 5 μL of isolated RNA was reverse transcribed and amplified using QuantiTect probe RT-PCR kit (Qiagen, Hilden, Germany) with sense primer (5′- GTGATCCATGTAAGCCCTCAGAA -3′), antisense primer (5′- GTCTGACATTGGGCTTTGAAGTTA-3′) and a TaqMan probe labeled with fluorophore and Quencher (5′-[6-FAM] AGGACCCCACATGTT [MGB CDPI3-BMN-Q535]-3′ (Jiménez-Clavero et al., 2006)). All oligonucleotides were ordered from Biomers, Ulm, Germany. The reaction was carried out in LightCycler^®^ 480 instrument (Roche, Penzberg, Germany). 10-fold serial dilutions of viral RNA isolated from purified WNV-Ita09 with defined titer served as standards for the quantification of viral genome copy numbers in mouse samples.

### 4.5 ELISA analysis

Microtiter plates (Nunc Polysorp^®^, ThermoScientific, Roskilde, Denmark) were incubated either with recombinant WNV proteins (WNV Ewt, WNV Equad, WNV EDIII) or viruses in coating buffer (35 mM Na_2_HCO_3_/15 mM Na_2_CO_3_, pH 9.6) in a total volume of 100 µL per well over night at 4°C. The coated amounts of proteins per well were 200 ng for the analysis with monoclonal antibodies and 300 ng for the analysis with mouse sera. The optimal coating amounts for recombinant proteins and virus particles were evaluated experimentally (data not shown). 10^5^ FFUs of virus particles were coated for the analysis with mouse sera. After three washing steps with 350 µL/well PBS 0.05% Tween20, plates were blocked for 2 h at room temperature (RT) with 5% non-fat milk powder in PBS (milk). After another wash step either mouse sera or monoclonal antibodies diluted in milk were incubated on the plates for 1.5 h at RT. Mouse sera were diluted 1:1,000 or 1:100 for binding to recombinant proteins or virus particles, respectively. The mouse monoclonal antibodies 4G2 (absolute antibody, Oxford, UK) and E16 (Millipore Merck, Darmstadt, Germany) were diluted 1:1,000 and 1:2,000, respectively. Following the third wash step, the anti-mouse IgG-HRP-conjugated antibody diluted in milk was incubated on the plates for another 1 h at RT. Subsequently to the final wash step, TMB substrate (Biozol, Hamburg, Germany) was incubated on plates for 30 min at RT in darkness, and the reaction was stopped with 1 M H_2_SO_4_. Absorbance was measured at 450 nm with 520 nm as reference in a microplate ELISA reader (TECAN, infinite M200 Basic, TECAN Austria GmbH, Grödig, Austria). Each sample was measured in duplicates in two independent experiments.

### 4.6 Focus reduction neutralization test

Focus Reduction Neutralization Test (FRNT) was performed as previously described (Finkensieper et al., 2022). In short, mouse sera taken three weeks after the second immunization were heat inactivated (56°C for 30 min), serially diluted in microplates and incubated with 75 FFU of purified WNV Ita09 for 1h at 37°C. The serum-virus-mixture was then added to Vero E6 cell monolayers in 96 well microwell plates and again incubated for 1 h at 37°C. Finally, the mix was removed and the cells were overlaid with 1% methylcellulose in DMEM with 2% FCS and 1% penicillin/streptomycin. After 16-18 h incubation at 37°C cells were fixed with 4% formaldehyde-PBS and Perm Wash buffer was used for permeabilization, blockage and washing of cells. The primary anti-flavivirus antibody 4G2 (absolute antibody, Oxford, UK, 1:2,000), an anti-mouse IgG HRP-conjugated secondary antibody (Dako, Denmark, 1:1,500) and TrueBlue peroxidase substrate (SeraCare, Milford, USA) were used for immunostaining. Spots were analyzed using the Immunospot Universal Analyzer (CTL, Cleveland, USA). The neutralizing antibody titer was defined as the reciprocal of the highest serum dilution that showed a minimal reduction in number of WNV foci of 50% compared to sera from the control group. Each serum was measured once in two independent experiments.

### 4.7 Statistics

Statistical analysis was performed with GraphPadPrism6 (Version 6.07, 2015). The ELISA data of sera on homologous recombinant proteins and the data of FRNT_50_ (arithmetic mean) assays were analyzed with Kruskal-Wallis Test. Data of ELISA assays using boost sera on virions were analyzed using two way ANOVA. All tests were followed by Dunn’s multiple comparisons test with *=p<0.05; **=p<0.01; ***=p<0.001. The survival rates were compared using Log-rank (Mantel-Cox) analysis.

## Data availability statement

The original contributions presented in the study are included in the article/supplementary material. Further inquiries can be directed to the corresponding author.

## Ethics statement

The animal study was approved by Landesdirektion Sachsen, Germany. The study was conducted in accordance with the local legislation and institutional requirements.

## Author contributions

RW: Investigation, Resources, Writing – original draft, Writing – review & editing, Data curation, Validation, Visualization. LI: Investigation, Resources, Validation, Visualization, Writing – original draft, Writing – review & editing. AR: Investigation, Writing – review & editing, Conceptualization. TG: Conceptualization, Investigation, Writing – review & editing, Funding acquisition, Resources, Supervision. JF: Conceptualization, Funding acquisition, Resources, Supervision, Writing – original draft. SU: Conceptualization, Funding acquisition, Resources, Supervision, Writing – original draft, Formal Analysis, Investigation, Project administration, Writing – review & editing, JF: writing - review & editing.
